# Drivers of disparities in stage at diagnosis among women with breast cancer: South African breast cancers and HIV outcomes cohort

**DOI:** 10.1371/journal.pone.0281916

**Published:** 2023-02-16

**Authors:** Witness Mapanga, Shane A. Norris, Ashleigh Craig, Oluwatosin A. Ayeni, Wenlong C. Chen, Judith S. Jacobson, Alfred I. Neugut, Paul Ruff, Herbert Cubasch, Daniel S. O’Neil, Ines Buccimazza, Sharon Čačala, Laura W. Stopforth, Hayley A. Farrow, Sarah Nietz, Boitumelo Phakathi, Tobias Chirwa, Valerie A. McCormack, Maureen Joffe

**Affiliations:** 1 Strengthening Oncology Services Research Unit, Faculty of Health Sciences, University of the Witwatersrand, Johannesburg, South Africa; 2 Department of Paediatrics, SAMRC/Wits Developmental Pathways to Health Research Unit, Faculty of the Health Sciences, University of the Witwatersrand, Johannesburg, South Africa; 3 Division of Medical Oncology, Department of Medicine, School of Clinical Medicine, Faculty of Health Sciences, University of the Witwatersrand, Johannesburg, South Africa; 4 Global Health Research Institute, School of Health and Human Development, University of Southampton, Southampton, United Kingdom; 5 South Africa Medical Research Council Common Epithelial Cancers Research Centre, Faculty of Health Sciences, University of the Witwatersrand, Johannesburg, South Africa; 6 National Cancer Registry, National Health Laboratory Service, Johannesburg, South Africa; 7 Sydney Brenner Institute for Molecular Bioscience, Faculty of Health Sciences, University of the Witwatersrand, Johannesburg, South Africa; 8 Herbert Irving Comprehensive Cancer Center, Vagelos College of Physicians and Surgeons, Columbia University, New York, New York, United States of America; 9 Department of Epidemiology, Mailman School of Public Health, Columbia University, New York, New York, United States of America; 10 Department of Medicine, Vagelos College of Physicians and Surgeons, Columbia University, New York, New York, United States of America; 11 Department of Surgery, Faculty of Health Sciences, University of the Witwatersrand, Johannesburg, South Africa; 12 Sylvester Comprehensive Cancer Center and Department of Medicine, Miller School of Medicine, University of Miami, Miami, Florida, United States of America; 13 Department of Specialized Surgery, Inkosi Albert Luthuli Central Hospital, Durban and Ngwelezane Hospital, University of KwaZulu-Natal, Empangeni, KwaZulu-Natal, South Africa; 14 Departments of Surgery and Radiation Oncology, Grey’s Hospital, University of KwaZulu-Natal, Pietermaritzburg, KwaZulu-Natal, South Africa; 15 Charlotte Maxeke Surgical Breast Unit, Charlotte Maxeke Johannesburg Academic Hospital, Johannesburg, South Africa; 16 School of Public Health, Faculty of Health Sciences, University of the Witwatersrand, Johannesburg, South Africa; 17 Section of Environment and Radiation, International Agency for Research on Cancer, Lyon, France; University of Wisconsin-Milwaukee Joseph J Zilber School of Public Health, UNITED STATES

## Abstract

**Objective:**

In low- and middle-income countries (LMICs), advanced-stage diagnosis of breast cancer (BC) is common, and this contributes to poor survival. Understanding the determinants of the stage at diagnosis will aid in designing interventions to downstage disease and improve survival from BC in LMICs.

**Methods:**

Within the South African Breast Cancers and HIV Outcomes (SABCHO) cohort, we examined factors affecting the stage at diagnosis of histologically confirmed invasive breast cancer at five tertiary hospitals in South Africa (SA). The stage was assessed clinically. To examine the associations of the modifiable health system, socio-economic/household and non-modifiable individual factors, hierarchical multivariable logistic regression with odds of late-stage at diagnosis (stage III-IV), was used.

**Results:**

The majority (59%) of the included 3497 women were diagnosed with late-stage BC disease. The effect of health system-level factors on late-stage BC diagnosis was consistent and significant even when adjusted for both socio-economic- and individual-level factors. Women diagnosed in a tertiary hospital that predominantly serves a rural population were 3 times (OR = 2.89 (95% CI: 1.40–5.97) as likely to be associated with late-stage BC diagnosis when compared to those diagnosed at a hospital that predominantly serves an urban population. Taking more than 3 months from identifying the BC problem to the first health system entry (OR = 1.66 (95% CI: 1.38–2.00)), and having luminal B (OR = 1.49 (95% CI: 1.19–1.87)) or HER2-enriched (OR = 1.64 (95% CI: 1.16–2.32)) molecular subtype as compared to luminal A, were associated with a late-stage diagnosis. Whilst having a higher socio-economic level (a wealth index of 5) reduced the probability of late-stage BC at diagnosis, (OR = 0.64 (95% CI: 0.47–0.85)).

**Conclusion:**

Advanced-stage diagnosis of BC among women in SA who access health services through the public health system was associated with both modifiable health system-level factors and non-modifiable individual-level factors. These may be considered as elements in interventions to reduce the time to diagnosis of breast cancer in women.

## Introduction

Breast cancer (BC) is currently the most diagnosed cancer in the world; among women, it accounts for close to 12% of all new cancers [1–3]. While survival is high in high-income countries (HICs), both incidence and mortality rates are increasing in low- and middle-income countries (LMICs) including South Africa (SA). These regions contribute over 53% of all new global BC cases and about 62% of global BC mortality [4]. In sub-Saharan Africa (SSA), the estimated 3-year survival of women diagnosed with breast cancer is around 58% as compared to 90% in the United States (US) [5, 6].

The high mortality rates among patients with BC are associated with a myriad of health system, patient, and environmental factors among the majority of the socio-economically disadvantaged populations of SSA. This group is subject to structural vulnerability, which is also true for socio-economically disadvantaged communities in HICs where disparities in BC survival rates are evident [7]. The socio-economically disadvantaged are more likely to experience poor general health status, disability, the simultaneous occurrence of more than one chronic condition or disease along with cancer (multimorbidity) and are less likely to access healthcare services [8, 9].

Late-stage at diagnosis is a major contributing factor to low survival in SSA [10]. A later stage at BC diagnosis means, by definition, that the tumour is of a larger size or has spread beyond the breast to regional lymph nodes (stage III) or distant metastatic sites (stage IV) [11]. Cultural, socio-demographic and health system challenges associated with disparities in stage at BC diagnosis among SSA populations are beginning to be reported in the literature [12–15]. We aimed to contribute further to these findings by performing an in-depth analysis of health system, socio-economic, and individual-level factors that may be associated with disparities in the stage at diagnosis of invasive BC in a cohort of socio-economically disadvantaged women from urban and rural communities enrolled in the South African Breast Cancer and HIV-outcomes (SABCHO) cohort [16]. We also assessed the complex hierarchical multi-level relationships among these (health system, socio-economic/household, and individual) factors to understand their roles in the stage of BC diagnosis.

## Methods

### Study setting and data source

South Africa (SA) has a dual (public and private) healthcare system. Most of the population, approximately 84%, depend on the resource-constrained public health care system, where public cancer diagnostic and treatment services are provided at no cost to patients who do however bear minimal transport and visit costs for hospital access [17]. Within this group, there exists immense inequality, a wide range of economic levels including poverty and high unemployment rates [17, 18]. The remaining 16% seek health services from the private sector (where services are paid for), aided by their private health insurance which will at least partially cover cancer diagnosis and treatment.

The SABCHO cohort study [16] has enrolled women with newly diagnosed invasive breast cancers at 5 tertiary public care hospitals in the Gauteng and KwaZulu-Natal (KZN) provinces of SA, with characteristics summarised in Table 1.

**Table 1:** Characteristics of the study public academic hospitals.

Tertiary academic hospitals*	Chris Hani Baragwanath Academic Hospital (CHBAH)	Charlotte Maxeke Johannesburg Academic Hospital (CMJAH)	Inkosi Albert Luthuli Central Hospital (Durban)	Grey’s Hospital (Greys)	Ngwelezane Hospital (Ngwelezane)
**Province**	**Gauteng**		**KwaZulu Natal**		
**Location**	Johannesburg	Johannesburg	Durban	Pietermaritzburg	Empangeni
**Catchment area population (N)**	Soweto (3 million)	Johannesburg East and Central (1.5 million)	Durban Metropolitan, KZN province (3.5 million)	Western KwaZulu-Natal (3.5 million)	Uthungulu, Umkhanyakude, Zululand (3 million)
**Urban / rural distribution**	Urban only	Urban only	Urban only	Mixed (urban and rural)	Rural only
**Patients referred from**	Mostly district primary care clinics; some regional hospitals	District primary clinics and regional hospitals	Regional hospitals	Regional hospitals	Regional clinics
**Core biopsy diagnostic procedure locations**	Mainly tertiary hospital	Mainly tertiary hospital	Mainly district secondary hospitals	Mainly district secondary hospitals	District secondary hospitals

*Cancer treatment is free in all the public hospitals in the South African Breast Cancers and HIV Outcomes Cohort study.

The Johannesburg hospitals serve predominantly urban and peri-urban communities, whereas the KZN sites serve both urban and rural communities. For the Chris Hani Baragwanath Academic Hospital (CHBAH) most BC patients are directly referred from local community-based primary care clinics in Soweto, Johannesburg. The other participating hospitals receive most of their patients from regional hospitals that receive symptomatic patients from their feeder district primary care clinics. In all these public sector settings, BC is diagnosed at a symptomatic stage because there are no mammography-based nor clinical breast examination population-based screening programmes in SA. The Johannesburg tertiary hospital breast units perform the diagnostic assessments for BC including core biopsies for histopathological examination, immunohistopathology and receptor subtyping and provide multimodal cancer treatments, whereas, in KZN, core biopsies are performed mainly in regional hospitals where breast cancer surgical procedures are often also performed, particularly for rural patients. Chemo- and radiation therapy and endocrine treatments are provided in all the tertiary hospital sites. Details of each site are summarised in Table 1.

### Participants recruitment and inclusion criteria

The SABCHO research team has prospectively collected data for 3,497 women with newly diagnosed invasive BC. Women were eligible to be recruited into the SABCHO study, if they were at least 18 years of age, were enrolled between July 1, 2015, and December 31, 2019, with a new diagnosis of stage I-IV invasive BC, had no prior history of cancer, received BC treatment at one of the study hospitals. A section of participants were clinically staged at the time of diagnosis using the 7^th^ edition of the American Joint Committee on Cancer (AJCC) BC. Trained staff at the hospitals collected through face-to-face interviews and entered the following data: socio-demographic information (e.g., age, marital status, employment status, the highest level of education completed); reproductive history (number of full-term pregnancies, use of oral and injectable contraceptives), and behavioural factors (alcohol consumption, smoking). Household or socio-economic scores were calculated using patients’ self-reported homeownership, home with indoor running water, car ownership, etc). Details of other collected variables and clinical data, and their calculations including distances from the place of residence to the place of diagnosis and knowledge of BC, have been reported previously [9, 13].

### Statistical analyses

**Fig 1:**
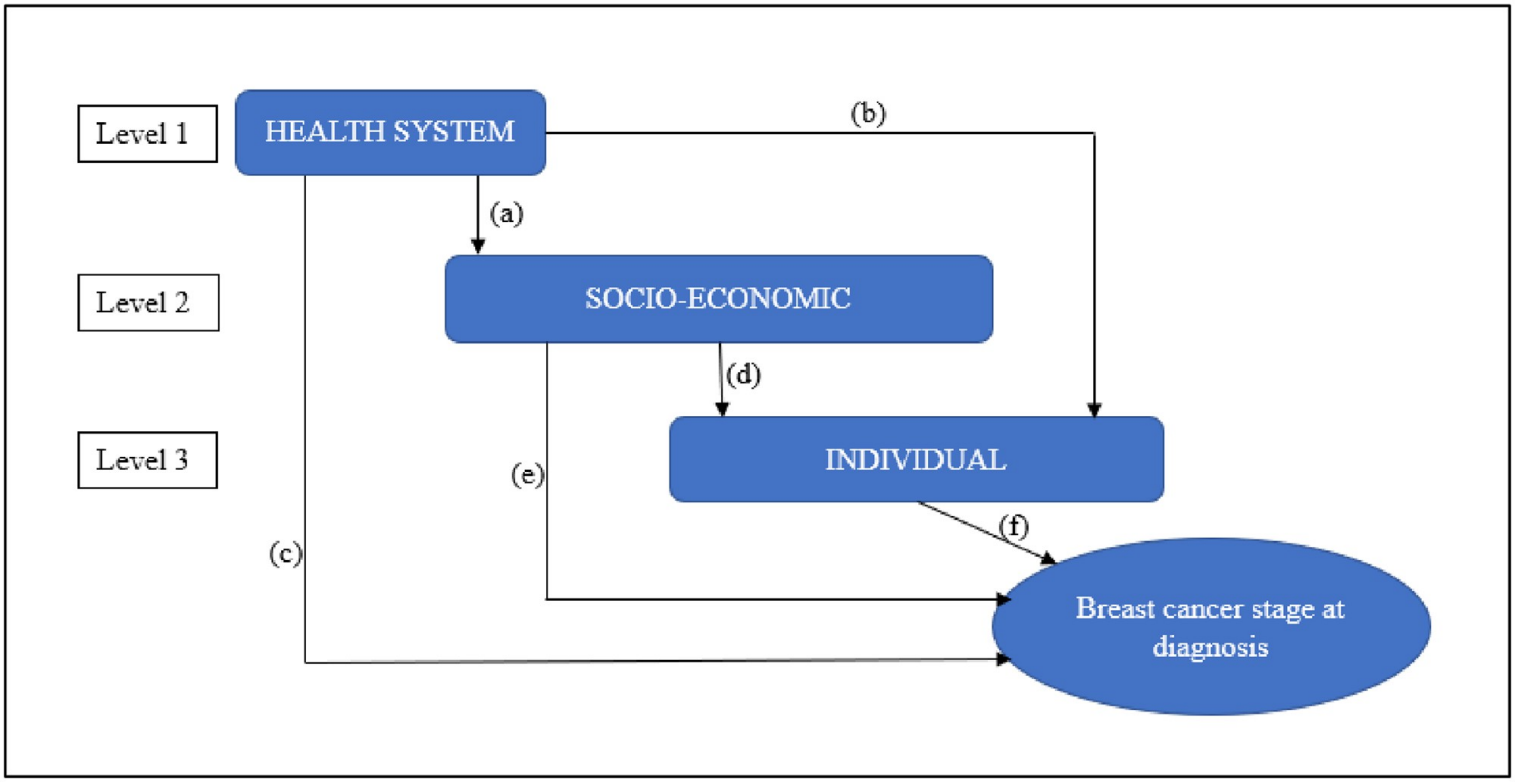
Conceptual hierarchical multi-level framework for factors potentially associated with breast cancer stage at diagnosis. *(a) health system affecting socioeconomic; (b) health system affecting the individual; (c) health system affecting breast cancer stage at diagnosis; (d) socioeconomic affecting the individual; (e) socioeconomic affecting breast cancer stage at diagnosis; (f) the individual affecting breast cancer stage at diagnosis.

We analysed the health system, socio-economic and individual-level characteristics of the cohort grouped by stage at diagnosis: early (stages I and II) and late (stages III and IV). We used Pearson chi-squared and Fisher’s exact tests to compare the distributions of values of categorical variables and the student’s t-test and the Wilcoxon rank-sum test to compare means and medians of continuous variables by stage group. We then conducted a hierarchical multivariate logistic regression analysis to examine the associations of individual, socio-economic, and health system-level characteristics with stage at diagnosis. This approach enabled us to investigate complex hierarchical multi-level relationships among the three categories, individual, socio-economic, and health system variables. Fig 1 describes our approach to the analysis of the hierarchical multi-level relationship among the three broad variables. The labelled arrows represent the multi-level relationship/ of the relevant variables with each other. Health system factors (the distal determinants) may affect the stage at diagnosis directly ((c), Fig 1), or indirectly ((a & b), Fig 1), via socio-economic and individual factors, which may, in turn, influence the stage at diagnosis.

Health system-level factors are wider determinants of health, such as access and referral routes to health care services and geographical location. These factors are not directly under individual control but are considered part of the environment in which people live and work. Health system-level factors were classified according to their geographical location (predominately urban, mixed (both urban and rural) or rural). Socioeconomic-level factors include wealth index, employment status and education attainment. This is because the level of education determines the level of state of a person’s employment status, and this has a bearing on the wealth index. Lastly, individual-level factors are non-socioeconomic and include age, knowledge, genetics, disease and comorbidity clinical characteristics (Table 2).

**Table 2:** Description of the three categories of individual, socio-economic, and health system variable.

Three Categories	Specific Factors/Variables within each Broad Category	Definitions and how variables were collected.
Health system level	Hospitals; access and referral routing for diagnosis	Hospitals/institutions were considered as part of the variables because each hospital embodies different characteristics such as its catchment area and population, its geographical location, and the health services offered.
		Referral routing was either ‘direct’ which means the patient was referred from primary care clinics directly to the tertiary hospital or ‘indirectly’ when patients are referred from primary clinics via secondary hospitals before they get to the tertiary hospitals. The name of the referring primary care clinic or secondary hospital was available on the referral forms.
Socio-economic level	Educational level; employment status; wealth index	Dedicated study nurses obtained patient socio-demographic data including educational level, employment status, reproductive history, smoking, alcohol use, family history of breast and other cancers, prior medical conditions, BC knowledge and attitudes, social support, entry into the health care system, sources of delay in obtaining care, and facilitators of access to care.
Individual level	Age; knowledge of BC; genetics; disease and clinical characteristics	
		Data on stage, grade, receptor status and treatment received were extracted from medical records.
		The wealth index was calculated based on self-reported home ownership, car ownership, possession of a washing machine or microwave, presence of a flush toilet inside the home and presence of indoor running water. Each item was assigned a score of 1 and the scores were added out of a total of six.
		Each patient’s residential address was documented at enrolment, and we computed the shortest straight-line distance to the enrolling hospital in kilometres (km) to define distance to the diagnosis hospital.

Model 1 of the hierarchical regression assessed the overall association of both health system factors with the BC stage at diagnosis ((c), Fig 1). This model excluded the socio-economic/household and individual variables. In model 2, all the socio-economic factors were added and their association with the BC stage at diagnosis was assessed, controlling for health-system factors (acting as confounders) ((a & e), Fig 1). The remaining effect of health system factors in model 2 thus reflected the part which was not confounded by socio-economic factors.

All the individual variables were added in model 3 to determine the association of personal and clinical characteristics with the stage at diagnosis, controlling for the health system, and socio-economic variables (both acting as confounders) ((a, d & f), Fig 1). The independent effects of the health system ((c), Fig 1), and socio-economic factors ((e), Fig 1) were determined.

We pooled aggregate data using the meta-analysis common-effect inverse-variance model to estimate the overall association of the three variable levels with BC stage at diagnosis. We also conducted subgroup analyses of the association of each of the three-block variables with the stage at diagnosis. We used Stata version 16 (StataCorp Ltd, Texas, US) to analyse these data.

### Ethical approval and consent to participate

The SABCHO study was approved by the University of the Witwatersrand Human Research Ethics Committee (Approval Number: Ml50351, dated: 6th May 2015, recertified M1911203 dated 28 January 2020), the University of KwaZulu-Natal Biomedical Research Committee, BF080/15, and the Institutional Review Board of Columbia University (protocol number AAAQ1359, dated 1st January 2016). The authors affirm that the research participants provided written informed consent for the publication of their de-identifiable data.

## Results

### Health system and socio-economic-level factors of women with stages I-IV breast cancer

In 3497 women with BC, 2058 (58.9%) were diagnosed with late-stage (stage III-IV) disease. The health system and socio-economic characteristics of these women are summarised and compared by stage in Table 3. Tertiary hospital site appeared associated with the stage at diagnosis and was included in subsequent hierarchical multivariate regression analyses. CHBAH had the greatest proportion of women presenting with early-stage disease (48.27%) as compared to Ngwelezane which had only 15.07%. In terms of late-stage disease, Ngwelezane had the greatest proportion (84.93%) as compared to CHBAH which had 51.73%. Overall, 63.86% (n = 1242) of women who were indirectly referred had late-stage diagnoses (p<0.001). At the CHBAH site, 39.0% of patients had been indirectly referred, compared with 57.0% at CMJAH, 74.0% at Durban, 93.0% at Greys and 88.0% at Ngwelezane. Those directly referred from primary care facilities received their core biopsies at the tertiary hospital pathology sites.

**Table 3:** Comparison analysis of the health system and socio-economic-level characteristics of women by breast cancer early and late-stage diagnosis in the South African breast cancers and HIV outcomes cohort.

	Early Stage		Late Stage		Total	p-value
	N	%	N	%	N (100%)	
Total	1439	41.1	2058	58.9	3497	
**HEALTH SYSTEM-LEVEL CHARACTERISTICS**						
**Nearby tertiary hospital (where patients were diagnosed)**						
CHBAH—Gauteng	656	48.27	703	51.73	1359	**<0.001**
CMJAH- Gauteng	332	38.16	538	61.84	870	
Durban	215	36.38	376	63.62	591	
Greys	225	37.25	379	62.75	604	
Ngwelezane	11	15.07	62	84.93	73	
**Referral routing for diagnosis**						
Direct from primary to tertiary	736	47.42	816	52.58	1552	**<0.001**
Indirect- primary to secondary to tertiary	703	36.14	1242	63.86	1945	
**SOCIO-ECONOMIC-LEVEL CHARACTERISTICS**						
**Highest level of education attained**						
Primary education or below	261	33.81	511	66.19	772	**<0.001**
Secondary education or higher above	1169	43.33	1529	56.67	2698	
**Employment status**						
Employed	402	42.05	553	57.95	956	**<0.001**
Unemployed	738	38.56	1176	61.44	1914	
Retired	295	47.81	322	52.19	617	
**Homeownership**						
Yes	899	42.09	1237	57.91	2136	0.158
No	536	39.67	815	60.33	1351	
**Household possession wealth index**						
1 (least wealthy)	201	28.76	498	71.24	699	**<0.001**
2	269	38.48	430	61.52	699	
3	290	41.43	410	58.57	700	
4	321	45.92	378	54.08	699	
5 (Wealthiest)	358	51.14	342	48.86	700	

Having less education (primary education and below) was associated with late-stage diagnosis as were unemployment and lack of household possessions (wealth index) (p<0.001 for all the above). Socio-economic factors that appeared to be associated with the stage at diagnosis and that were subsequently explored in hierarchical regression analyses were the highest education level attained (p <0.001), employment status and household possession wealth index (p<0.001).

### Individual-level factors of the women with stages I-IV breast cancer

As Table 4 shows, the mean age of the SABCHO participants was 55.7 (SD 14.3) years at diagnosis. Those women diagnosed at an early stage were older, than those diagnosed late (p = 0.006) and a larger proportion were postmenopausal (p = 0.001). Married women, those with a family history of cancer, and those with good knowledge of cancer were more likely than others to have been diagnosed at an early stage. Most women were diagnosed with hormone-responsive tumours, luminal A (ER/PR+/HER2-) (61.0%) and luminal B (ER/PR+/HER2+) (16.5%). The majority of women (70.6%) were diagnosed within 3 months of first recognising their breast symptoms and close to 58.7% (n = 2051) of women resided within 20km from their pathology diagnostic facility. Individual-level factors that initially appeared to be associated with later stage at diagnosis and that were subsequently explored in hierarchical regression analyses included younger age at diagnosis (p = 0.006), more full-term pregnancies (p<0.001), single marital status (p = 0.001), self-reported family history of breast cancer (p<0.001), less knowledge of breast cancer prior to diagnosis (p = 0.034), higher body mass index at diagnosis (p = 0.016), current cigarette smoker (p<0.001) and regular alcohol intake (p = 0.005). Other individual factors that appeared to be associated with later stage at diagnosis were greater residential distance to histopathology diagnostic hospital (p<0.001) and longer delay period from initial symptom detection to immunohistopathology confirmed diagnosis (p<0.001). Clinical factors that initially appeared to be associated with later stage at diagnosis were self-reported comorbid diabetes (p = 0.024), hypertension (p = 0.001), HIV status (p<0.001), BMI (p = 0.016) and BC receptor subtype (p<0.001).

**Table 4:** Comparison analysis of the individual characteristics of women by breast cancer early and late-stage diagnosis in the South African breast cancers and HIV outcomes cohort.

	Early Stage		Late Stage		Total	p-value
	N	%	N	%	N (100%)	
Total	1439	41.1	2058	58.9	3497	
**INDIVIDUAL CHARACTERISTICS**						
**Age at diagnosis (mean(sd)), years**						
	1439	56.65(13.71)	2058	54.95(14.69)	3497 [55.65(14.32)]	**0.006**
**Age at menarche (mean(sd)), years**						
	1332	15.94 (2.49)	1896	14.68 (2.29)	3228[15.20(2.38)]	0.2674
**Number of complete/full-term pregnancies (median (interquartile range))**						
	1307	3(2–4)	1907	3(2–4)	3214[3(2–4)]	**<0.001**
**Menstrual status**						
Pre-menopausal	499	37.55	830	62.45	1329	**0.001**
Post-menopausal	940	43.36	1228	56.64	2168	
**Marital status**						
Single	368	36.15	650	63.85	1018	**0.001**
Married/cohabiting	582	43.47	757	56.53	1339	
Divorced/widowed	485	42.92	645	57.08	1130	
**Family history of cancer**						
Yes	241	49.59	245	50.41	486	**<0.001**
No	1167	39.86	1761	60.14	2928	
**Used contraception**						
Yes	872	40.86	1262	59.14	2134	0.630
No	564	41.69	789	58.31	1353	
**Knowledge of breast cancer**						
Less/Intermediate knowledge	393	38.49	628	61.51	1021	**0.034**
Good knowledge	1049	42.37	1427	57.63	2476	
**Body mass index (kg/m** ^**2**^ **)**						
<25	198	36.07	351	63.93	549	**0.016**
25–29.9	368	43.60	476	56.40	844	
≥30	802	41.84	1115	58.16	1917	
**Smoke cigarettes**						
Yes	224	51.61	210	48.39	434	**<0.001**
No	1211	39.67	1842	60.33	3053	
**Alcohol intake**						
Yes	309	45.91	364	54.09	673	**0.005**
No	1126	40.00	1688	60.00	2814	
**Diabetes**						
Yes	208	46.02	244	53.98	452	**0.024**
No	1227	40.43	1808	59.57	3035	
**Hypertension**						
Yes	633	44.48	790	55.52	1423	**0.001**
No	802	38.86	1262	61.14	2064	
**HIV**						
Negative	1146	42.76	1534	57.24	2680	**<0.001**
Positive	273	35.73	491	64.27	764	
**HIV positives on antiretroviral therapy at the time of breast cancer diagnosis**						
Yes	228	37.38	382	62.62	610	0.082
No	45	29.80	104	70.20	151	
**Receptor subtype**						
ER/PR+/ HER2-	971	45.80	1149	54.20	2120	**<0.001**
ER/PR/ HER2+	202	35.25	371	64.75	573	
ER/PR-/HER2+ (HER2 Enriched)	69	29.61	164	70.39	233	
ER/PR/HER2- (Triple-Negative)	196	35.64	354	64.36	550	
**Distance for the individual to travel to the closest diagnosis hospital**						
<20 km	898	43.78	1153	56.22	2051	**<0.001**
20 to 59 km	358	37.41	599	62.59	957	
60 to 100 km	42	30.22	97	69.78	139	
>100 km	141	40.29	209	59.71	350	
**Time (in months) from identifying the problem to histopathology-confirmed diagnosis**						
< = 3	1118	45.28	1351	54.72	2469	**<0.001**
>3	324	31.52	704	68.48	1028	

### Health system-level characteristics, and later stage at diagnosis (Table 5, model 1)

**Table 5:** Hierarchical multiple logistic regression models of the associations between community, socio-economic, individual-level characteristics, and stage at diagnosis among the 3497 women in the South African breast cancers and HIV outcomes cohort.

Variables	Health system characteristics model	Health system + socio-economic/household model	Health system + socio-economic/household + individual model
	OR^1^ (95% CI)	OR^2^ (95% CI)	OR^3^ (95% CI)
**HEALTH SYSTEM-LEVEL CHARACTERISTICS**			
**Tertiary hospital**			
CHBAH—Gauteng	1	1	1
CMJAH- Gauteng	**1.52 (1.28–1.81)**	**1.64 (1.37–1.97)**	**1.70 (1.37–2.11)**
Durban	**1.44 (1.71–1.77)**	**1.45 (1.17–1.79)**	**1.92 (1.39–2.66)**
Greys	**1.28 (1.03–1.58)**	1.12 (0.90–1.40)	1.26 (0.94–1.69)
Ngwelezane	**4.40 (2.28–8.46)**	**3.00 (1.54–5.85)**	**2.89 (1.40–5.97)**
**Referral routing for diagnosis**			
Direct from primary to tertiary	1	1	1
Indirect- primary to secondary to tertiary	**1.49 (1.28–1.73)**	**1.36 (1.16–1.59)**	**1.32 (1.07–1.62)**
**SOCIO-ECONOMIC-LEVEL CHARACTERISTICS**			
**Highest level of education attained**			
Primary education and below		1	1
Secondary education and above		**0.78 (0.65–0.93)**	0.82 (0.65–1.01)
**Employment**			
Employed		1	1
Unemployed		1.05 (0.89–1.24)	1.14 (0.94–1.40)
Retired		0.84 (0.68–1.04)	1.12 (0.84–1.50)
**Homeownership**			
Yes		1	1
No		1.04 (0.90–1.21)	1.03 (0.85–1.25)
**Wealth index**			
1		1	1
2		**0.77 (0.61–0.97)**	0. 83 (0.64–1.08)
3		**0.69 (0.55–0.88)**	0.84 (0.64–1.11)
4		**0.55 (0.43–0.69)**	**0.72 (0.54–0.95)**
5 (Wealthiest)		**0.48 (0.38–0.62)**	**0.64 (0.47–0.85)**
**INDIVIDUAL CHARACTERISTICS**			
**Age at diagnosis**			
			0.99 (0.98–1.00)
**Age at menarche**			
			1.02 (0.98–1.06)
**Number of complete/full-term pregnancies**			
			**1.07 (1.02–1.13)**
**Menstrual status**			
Pre-menopausal			1
Post-menopausal			0.99 (0.75–1.31)
**Marital status**			
Single			1
Married/cohabiting			0.88 (0.71–1.09)
Divorced/widowed			0.87 (0.69–1.09)
**Family history of cancer**			
No			1
Yes			**0.77 (0.61–0.97)**
**Knowledge of breast cancer**			
Less/Intermediate knowledge			1
Good knowledge			**0.79 (0.66–0.95)**
**Body mass index (BMI)**			
<25			1
25–29.9			0.82 (0.63–1.07)
≥30			0.93 (0.73–1.18)
**Smoke cigarettes**			
No			1
Yes			**0.67 (0.52–0.87)**
**Alcohol intake**			
No			1
Yes			0.84 (0.68–1.05)
**Diabetes**			
No			1
Yes			0.92 (0.71–1.19)
**Hypertension**			
No			1
Yes			0.86 (0.71–1.05)
**HIV**			
Negative			**1**
Positive			0.97 (0.78–1.22)
**Receptor subtype**			
ER/PR+/ HER2-			1
ER/PR+/ HER2+			**1.49 (1.19–1.87)**
ER/PR-/HER2+ (HER2 Enriched)			**1.64 (1.16–2.32)**
ER/PR/HER2- (Triple-Negative)			**1.36 (1.08–1.71)**
**Distance from residence to diagnostic hospital**			
<20 km			1
20 to 59 km			1.11 (0.89–1.38)
60 to 100 km			1.00 (0.70–1.42)
>100 km			0.85 (0.63–1.15)
**Time (in months) from identifying the problem to the first health system entry**			
< = 3			1
>3			**1.66 (1.38–2.00)**

As Table 5 shows, health system-level characteristics were the most important risk factors for late-stage BC diagnosis in the study population; both hospital where patients were enrolled in SABCHO and referral routing for diagnosis were associated with late-stage in all three hierarchical multivariate regression models. In the core health system level characteristics model 1, women diagnosed at Ngwelezane hospital, were more than 4 times more likely to have late-stage BC than women diagnosed at CHBAH hospital. Larger proportions of women diagnosed at the other hospitals had late-stage BC. Having been referred from feeder primary clinics to secondary hospitals and then to the tertiary hospitals was also associated (OR = 1.49 (95% CI: 1.28–1.73)) with late-stage BC diagnosis.

### Health system- and socio-economic-level characteristics, and later stage at diagnosis (Table 5, model 2)

The associations of socio-economic-level factors with the stage at diagnosis were adjusted for confounding by health system factors in model 2. Two socio-economic variables, attaining a secondary and higher level of education compared with primary or lower education and increasing wealth index, respectively for wealth indices 2–5, compared with poorest wealth index 1, were associated with earlier stage at BC diagnosis. The associations of health system-level factors with stage were attenuated but remained statistically significant in model 2. Notably, after adjusting for socio-economic factors, the later stage at diagnosis originally seen in Greys and Ngwelezane hospitals still existed but was now attenuated [Greys site (OR = 1.12 (95% CI: 0.90–1.40)), and Ngwelezane site (OR = 3.00 (95% CI: 1.54–5.85))].

### Health system, socio-economic and individual-level characteristics, and later stage at diagnosis (Table 5, model 3)

In model 3, higher parity (number of complete or full-term pregnancies, receptor subtype [luminal B, HER2 enriched and Triple Negative subtypes] and taking more than 3 months from identifying the BC problem to first health system entry (OR = 1.66 (95% CI: 1.38–2.00))), were associated with late-stage diagnosis, even when both health system- and socio-economic-level factors were taken into account. Other individual-level factors such as having a family history of cancer (OR = 0.77 (95% CI: 0.61–0.97)), having good knowledge of BC (OR = 0.79 (95% CI: 0.66–0.95)) and smoking cigarettes (OR = 0.67 (95% CI: 0.52–0.87)) also were associated with an early-stage diagnosis of BC in the presence of both health system and socio-economic-level factors. The health system-level factors remained strongly associated with late-stage BC diagnosis.

The health system-level (tertiary hospitals) characteristics continued to be positively associated with late-stage BC diagnosis (percentage late-stage increased from hospitals that predominantly serve urban distribution to those that serve rural distribution), whilst wealth index characteristics showed a protective effect on being diagnosed with late-stage BC (percentage late-stage decreased with increase in wealth index) even when adjusted for each other and age at diagnosis (S1 Fig).

## Discussion

In this study, we examined the associations of the health system, socio-economic (household) and individual factors with BC stage at diagnosis in a cohort of women. We identified health system-level factors (tertiary hospital and referral routing for diagnosis) and individual-level factors (having more full-term pregnancies and breast receptor subtypes) as important correlates of late-stage at BC diagnosis. We found that greater wealth was protective against late-stage BC at diagnosis. Overall, health system-level variables, tertiary hospital and referral mode for diagnosis, were the most important risk factors for late-stage BC diagnosis.

In the presence of both health system and individual-level factors, we found that women who were relatively wealthier, and better educated were more likely to be diagnosed with BC earlier. These findings are congruent with what was identified in other studies that have been conducted in LMIC settings [12, 13, 19–21]. There is a high possibility that having higher education might mean having good knowledge of BC because in our study, having good knowledge of BC was found to be protective of later-stage diagnosis of BC. This link between higher education and having good knowledge of BC was also explained in a Nigerian study [20]. Increasing BC awareness through health promotion and education should be prioritised regardless of socio-economic status and such activities might be beneficial if introduced early in the mainstream education system and through sustained community-level health and cancer literacy interventions.

In SSA the prevalence of prolonged delays of more than three months from symptom recognition to care-seeking can range up to 70% as compared to less than 17% in the US and Europe [22]. Furthermore, BC progression is time-dependent [12, 23]. Our findings have shown the significance of delaying for more than 3 months, from identifying BC symptoms/problems to first health system entry, as a predictor of advanced-stage diagnosis. This finding is consistent with what has been reported in West Africa and in the ABC-DO cohort of 1795 women from SSA with breast cancer that included women from our Soweto site as well as women from Namibia, Uganda, Zambia and Nigeria [14, 24]. Reducing the interval between identifying the BC problem and first health system entry is crucial to downstaging disease at diagnosis and this can be facilitated by bringing BC diagnosis services into the community or primary care facilities with for example screening clinical breast examination, fine-needle aspiration point-of-care triaging diagnostic approaches and by simplifying the referral process (as suggested by our findings). Such early detection strategies have been demonstrated to work in other parts of Africa [25, 26] and in a 20-year Indian trial, proven to downstage disease among women of all ages and to increase survival among postmenopausal women [27].

In the model with health system and socio-economic factors, luminal B, HER2 enriched, and triple-negative BC subtypes were found to be significantly associated with later-stage diagnosis of BC in our cohort when compared to luminal A. This is similar to what has been found elsewhere in Africa and high-income countries, for these more aggressive, faster-growing tumours [6, 14, 28].

### Study strengths

The SABCHO is a large prospective study (with few loss-to-follow-ups) covering two of SA’s biggest provinces and has detailed data on several patient and tumour characteristics. Our study has generated important information on the impact of the health system, socio-economic, and individual-level factors on invasive BC stage at diagnosis among socioeconomically disadvantaged women from urban and rural communities, which we can generalise to the rest of the country’s provinces.

### Study limitations

Although a lot of our patient variables were objectively measured, a few of the variables were self-reported, such as educational level, wealth status, and distance to the diagnostic hospital. There is a chance that information bias would have occurred from some of these variables and this might likely have resulted in over or underestimating their true effect. Furthermore, there is likely to be a potential selection bias within the cohort we used because those enrolled might not have been representative of the women diagnosed with breast cancer at all the hospital sites. This means the reported estimates among the various variables as well as generalisation of the results to the rest of the women with breast cancer, need to be done cautiously.

## Conclusion

Late-stage diagnosis of BC among predominantly poor women in SA who access health services through the public health system is associated with health system-level factors as well as individual-level factors. Having shown the hierarchical interrelation between health system, socio-economic and individual factors, addressing community health systems barriers by introducing affordable evidence-based community and primary care screening and developing inexpensive point-of-care diagnostic tests may facilitate downstaging of BC in most poor-resourced settings. The strong association of late-stage diagnosis with an indirect referral for diagnosis of women at regional secondary hospitals needs urgent examination. The CHBAH site which receives the majority of its patients directly referred from primary healthcare clinics in Soweto had the greatest proportion of women presenting with early-stage disease, which suggests that secondary hospitals should not be involved in cancer diagnosis and management in South Africa. Before advocacy for any policy change is made, however, more research is required. We need to unpack in detail, the associations between referral mode and (i) women’s delay periods before accessing care and (ii) the number of visits and time spent within the referral facilities from the date of first access to the date of definitive diagnosis. Qualitative research with all stakeholders to understand in detail the reasons for patient and health system delays also need to be undertaken. We also need to understand how best to intervene in communities and in primary care settings to implement existing policies and guidelines for screening clinical breast examinations.

## Supporting information

S1 Fig Tertiary hospital and wealth index with a late-stage BC diagnosis. (DOCX)

S1 Data Minimal dataset. (XLSX)
