# Supplementary figures and images for: Drivers of disparities in stage at diagnosis among women with breast cancer: South African breast cancers and HIV outcomes cohort
Source: PLoS One. 2023 Feb 16;18(2):e0281916. doi: 10.1371/journal.pone.0281916 (PMC9934316; doi:10.1371/journal.pone.0281916)

**Supplementary Figure**


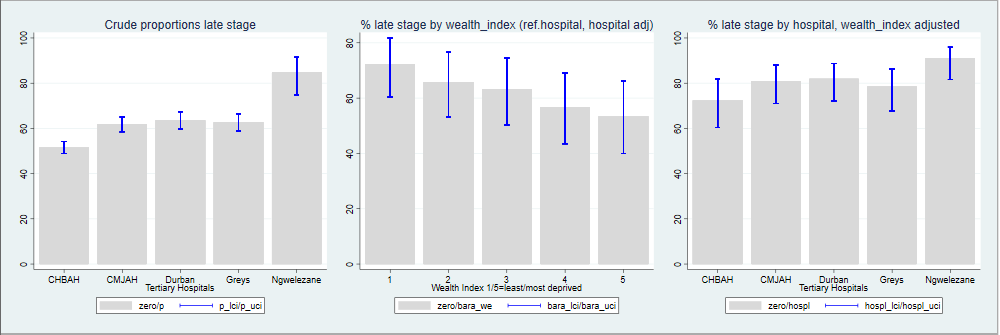


***S1 Fig: Tertiary hospital and wealth index with a late-stage BC diagnosis***

Supplement: S1 Fig — (DOCX) [file pone.0281916.s001.docx]
